# Surnames and ancestry in Brazil

**DOI:** 10.1371/journal.pone.0176890

**Published:** 2017-05-08

**Authors:** Leonardo Monasterio

**Affiliations:** 1 Department of Regional, Urban and Environmental Studies, Institute for Applied Economic Research, Brasília, DF, Brazil; 2 Graduate School of Economics, Universidade Católica de Brasília, Brasília, DF, Brazil; Universitat Pompeu Fabra, SPAIN

## Abstract

This paper presents a method for classifying the ancestry of Brazilian surnames based on historical sources. The information obtained forms the basis for applying fuzzy matching and machine learning classification algorithms to more than 46 million workers in 5 categories: Iberian, Italian, Japanese, German and East European. The vast majority (96.7%) of the single surnames were identified using a fuzzy matching and the rest using a method proposed by Cavnar and Trenkle (1994). A comparison of the results of the procedures with data on foreigners in the 1920 Census and with the geographic distribution of non-Iberian surnames underscores the accuracy of the procedure. The study shows that surname ancestry is associated with significant differences in wages and schooling.

## Introduction

Official census surveys in Brazil do not register information on the population’s ancestry. Traditionally censuses have used just five categories to determine color/race, namely, black, white, mixed, yellow and native Brazilian [Amerindian]. (IBGE, the Brazilian Statistical Office, uses the term “color/race”. We acknowledge that “race” has no biological meaning. In this paper, we use this expression as a way to follow the national standard.) Although those categories do have social significance, they are often far too broad to allow for specific applications such as socioeconomic or epidemiological studies.

This article contributes to the classification of the ancestry of Brazilian surnames. It also innovates by using historical databases to associate surnames to ancestry and by applying machine learning algorithms to classification. To obtain the contemporary distribution of surnames, the study made use of the 2013 Annual Social Information Report (Relação Anual de Informações Sociais) hereafter referred to as the RAIS [1]. The database is a very large restricted-access administrative file that contains 46.8 million observations on all Brazilians workers in the formal labor market.

There is a fairly robust literature on classification of surnames [2][3][4][5]. Concerning the applications of the classification of surnames, there are papers in epidemiology [6][7] and contributions in the field of Political Science [8] and of Anthropology [9]. In biodemography, methods based on surnames have been widely applied in order to infer genetic profiles of populations [10][11][12]. Some more recent studies have used names to investigate long term social themes: analysis the spatial distribution of families [13]; investigation of social mobility [14][15][16]; and the use of first names to study immigrant assimilation in the United States [17]. There is no record of any Brazilian study addressing surname classification.

This paper shows the potential of using historical datasets of immigrants for classification of surnames. The recent boom of freely available on-line archives of historical data, such as the USA Census [18], provides valuable information for researchers in this field. Contemporary datasets on surname frequency run the risk of containing data on immigrants and their descendants. For instance, MOHAMED, BOSCH, SINGH and CHEN are among the 500 most common surnames in contemporary Spain. This possible bias is reduced when historical sources are used. Another advantage is that databases of immigrants sometimes already contain changes in the spelling of surnames that will be passed on to their descendants.

Only five immigrant groups were considered: Iberian (Spaniards and Portuguese); Italian; German; East European and Japanese, reflecting the main countries of origin of immigrants that came to Brazil after 1872 [19]. In Brazil’s case, it is inappropriate to use surname methods to identify the ancestry of native Brazilians or Afro-brazilians descendants because they have adopted or rather, have been forced to adopt Iberian surnames. Thus the term “ancestry”, as used here, refers to the ancestry of the surname rather than that of the individual. Nevertheless, those two groups are approximately covered by the classifications ‘black’, ‘mixed’ and ‘native’ used by the IBGE ([20] and [21] discuss color and race in the Brazilian censuses).

Thus the method being proposed here should be looked on as a complement to the color/race classification and not a potential replacement for it. As will be shown below, there are glaring differences of income and schooling levels among the classes of ancestry that would not have remained undetected if the analysis had been restricted to the official color/race categories.

## Materials and methods

### Data

The main historical sources of migrants’ ancestry were the registers of the Immigration Museum (Museu da Imigração) in São Paulo which were obtained by webscrapping [22], historical data on the interstate slave trade [23] and from the micro-data of the North American censuses [18]. Other historical sources and databases with lists of names were also considered. The complete list of sources is set out in Table 1 and details are provided in the S1 Appendix. As can be seen, the study was built based on more than 5 million registrations.

**Table 1:** Description of sources of names and ancestry data.

Source	Nationality	Number of observations
USA Census 1880 and 1910 [18]	Various	4,715,496
Fragoso and Ferreira [23]	Iberian	63,818
Museu da Imigração [22]	Various	186,193
Common Japanese Surnames [24]	Japanese	4,000
Heraldica de Apellidos Españoles [25]	Iberian	5,138
Frecuencias de apellidos [26]	Iberian	500
Banco de sobrenomes—cidadania italiana [27]	Italian	6,573
Emigrazione Veneta [28]	Italian	60,889
Total	Various	5,042,107

As expected the sources contain transcription and writing or typing errors, especially in the names of immigrants gleaned from the North American censuses, and these have affected the accuracy of the algorithms. Thanks to the initial volume of information however, it was possible to apply rigorous criteria and exclude surnames that only appeared very rarely in the census micro-data. Whenever, in the historical sources, the same surname was attributed to more than one category, the following steps were taken: 1) in the same data bank, keep the names that have more than 90% of the registrations associated to the same ancestry; 2) in the United States census micro-data, remove surnames that appear less than eight times; 3) give priority to the information of the São Paulo Immigration Museum. By removing repetitions, non-analyzed nationalities and errors, it was possible to obtain 71,791 surname-ancestry pairs.

That base was the point of departure for the fuzzy matching and machine learning processes described in the sections that follow below. Table 2 presents the distribution of surnames according to ancestry.

**Table 2:** Distribution of surname-ancestry in the reference data.

Ancestry	Acronym	Number of observations
Iberian	IBR	10,142
Italian	ITA	26,191
German	GER	22,502
Japanese	JPN	5,375
East European	EAS	7,581
Total		71,791

In the case of contemporary names, the two surnames for each individual were obtained from the RAIS database. Most people in Brazil have two surnames inherited from the mother and the father, in that order. Furthermore, people that get married may or may not adopt the surname of the other party but traditionally women drop the surname of the mother and add that of the husband. In order not to lose information it was decided to consider the two surnames of each individual (whenever they existed). The analysis of two surnames distinguishes the present work from other papers. Non-significant particles were excluded and so were first names. The algorithm for identifying and removing compound first names is explained in S2 Appendix. That procedure resulted in around 530,000 unique surnames.

The five commonest surnames in Brazil (SILVA, SANTOS, OLIVEIRA, SOUZA, PEREIRA) account for 21 million of the 46.8 million names registered in the RAIS, that is to say, 45% of the registrations in that database. On the other hand, the number of names that only occurred once in the base was over 204,000. In spite of the overall quality of the RAIS data, a simple visual inspection shows that most of those cases are simple typing errors. The techniques that will be described in the next section were designed to overcome this last problem.

### Methods

#### Overview

**Fig 1:**
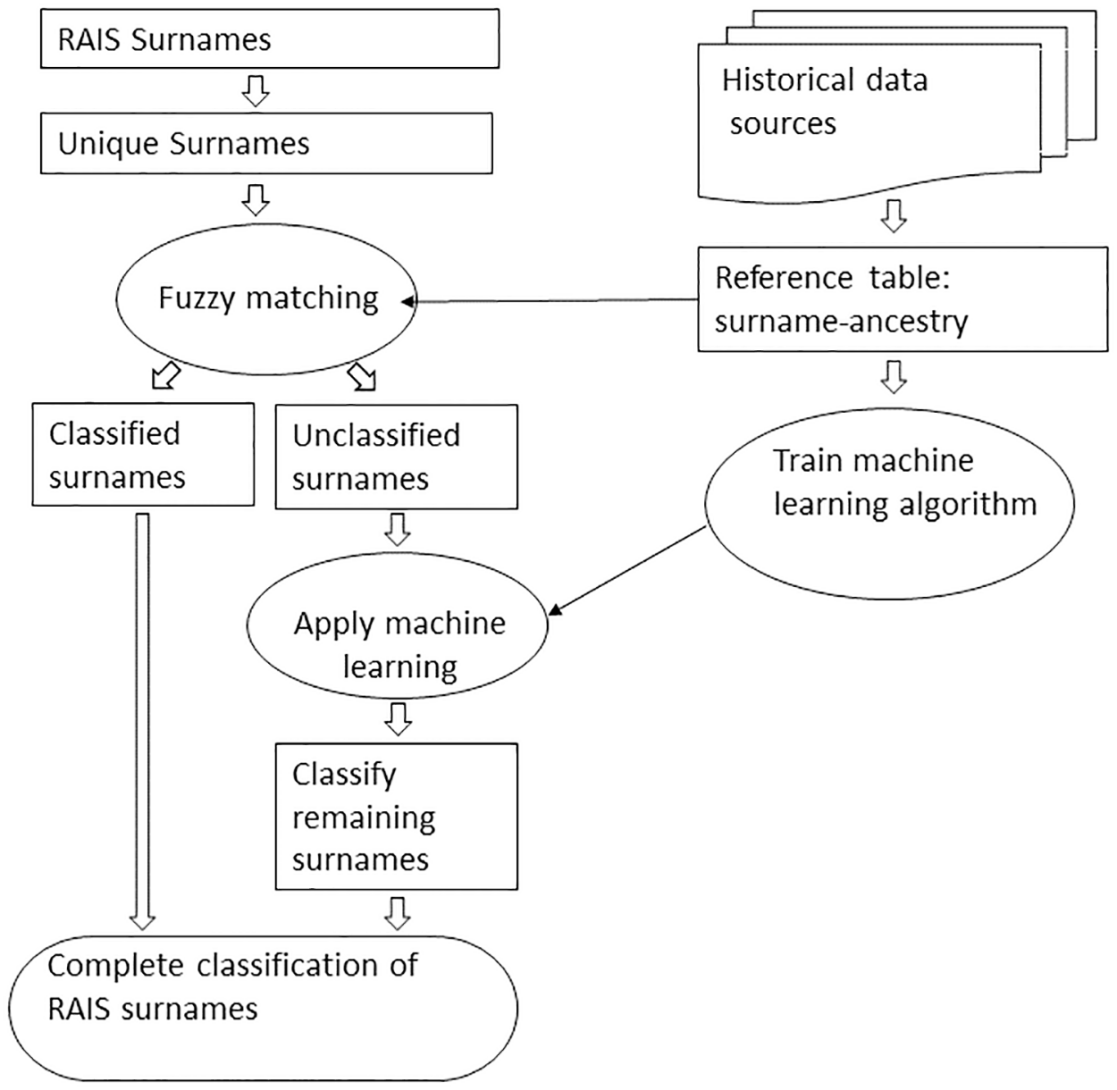
Flow diagram of surname classification.

Fig 1 shows the classification process. Based on both historical and contemporary data, a reference document is created which associates surnames to ancestry. This file has a double function: to serve as the base for fuzzy matching, and to serve as the base for the application of the two machine learning algorithms.

As the figure shows, the second step is to establish the correspondence between the reference table and the surnames, using fuzzy matching. Not all of the names were classified in this initial stage because many of them are not sufficiently similar to those in the reference table. Thus, only the surnames without any correspondence will be subjected to the application of the machine learning algorithms and these latter algorithms have been trained on the basis of the aforementioned reference table. Once all the unique surnames have been associated to an ancestry, then the RAIS database is used once more to classify the names of all the individuals. The subsections that follow below give details of the steps involved. All the analyses were made using R 3.2.2 [29] and its packages [30][31][32][33][34].

#### Fuzzy matching

Fuzzy matching methods make it possible to associate two sequences of characters or strings even when they are not identical. In this application, very conservative techniques were adopted to ensure there would be no mistaken matches of surnames in the reference table with those in the RAIS database. The criterion applied was Optical String Alignment (OSA) which matches strings when the distance between them is equal to or less than a predetermined value. Distance, in this context, means the number of changes (insertions, exclusions or changes of position) that are needed for the strings to become identical. The algorithm seeks out the perfect matches first—that is where the distance is zero—and only after that the search is made for distance values higher than zero. As an example, the names MUELLER and MILLER are fuzzy matched if d = 2 in the OSA criterion. If the value is set at d = 1 however, which was the value set in this research, they do not match.

#### Cavnar and Trenkle

The text categorization method proposed by Cavnar and Trenkle [2] is simple, requires little computing power and has been used in a variety of applications in the classification of languages and surnames. The algorithm can be synthesized in the following steps:

1. Create n-grams from the set of words with their categorization already known. This is called the training set. N-grams are sequences with a length n formed from the words. For example, the surname “LIMA” would have the following 3-grams: “_ _ L”; “_LI”; “LIM”; “IMA”; “MA_”, “A_ _”. The spaces before and after the word were added to preserve the information on the beginning and end of the surname. In this study, it means forming n-grams for all the surnames associated to each one of the ancestries;
2. Organize a table with n-gram frequencies in decreasing order for each one of the categories. Thus the highest ranking n-grams will be those that are more frequent in each one of the categories. This table of profiles serves as the base for classifying new names into categories;
3. Create n-grams of the new surnames which are then compared with each one of the rankings of the categories that were created in step 2;
4. Calculate the total distance or, as the authors of the method call it, the out of place measure, between the word and the ranking of the n-grams of each category. The total distance is the sum of the positions that the n-grams of the names outside the training set occupy in the rankings of profiles obtained in step 2;
5. Designate the category with the least total distance in relation to the profiles, as the category for the new surname. When implementing Cavnar and Trenkle’s algorithm it is necessary to define the value of n of the n-grams and also how many of those elements will compose the ranking. In this paper, the n in the n-gram was set to 3 and 1,250 n-grams were considered altogether. Those values were chosen because they provided the highest degree of accuracy in the classification. There is no objective rule for choosing these values. Several combinations of the size of n-grams and their number were tried and we chose the one that obtained the highest accuracy

#### Naïve Bayes

The Naïve Bayes classification algorithm is also based on the patterns of n-gram distribution in accordance with the categories. It is the most used classification because, despite its simplicity, it tends to show surprisingly accurate results. The idea is to apply Bayes rule to obtain the probability of a given surname’s pertaining to a class conditioned by its n-grams. In formal terms, given a set of surnames associated to y classes the classification s given by:

$$\begin{array}{c} c=argmaxP(y|s)P(s) \end{array}$$ (1)

In other words, each surname is classified a posteriori according to the greatest probability. Usually, the a priori distribution is based on the shares of classes in the training set.

The term naïve in the name of the method refers to the presupposition that the probabilities of the occurrences of the n-grams. That is obviously false Even so, the Naïve Bayes method performs very well in text classification applications, even in comparison with other methods that dispense with the independence hypothesis [35].

## Results

### Fuzzy matching results

The fuzzy matching process made it possible to classify the majority of individuals. Although only 293,634 of the 531,009 unique surnames found in the RAIS data were identified by fuzzy matching, the number corresponds to 96,7% of the workers. That is because the identified names are far more popular than those that were not identified. It should be noted that this result was obtained even with the adoption of the conservative option to attribute a value of 1 to the maximum distance in the Optimal String Alignment (OSA) algorithm The 3.3% of individuals in the RAIS whose names were not classified by the fuzzy matching were classified by the machine learning algorithm. Even though the percentage of non-classified individuals is relatively small, it is worthwhile applying such methods when the objective is to identify non-Iberian immigrants, because they may very well be over-represented in that group.

### Accuracy of machine learning

The first step consisted of dividing up the data into the training set and the test set. In this case the process started with the exact matches table (distance = zero) of pairs of surnames with ancestries and the same was done with those that occurred in the RAIS base (38,030 registrations). The training dataset is available on S1 Data. 20% of such pairs were separated for testing. The calibrated algorithms were later applied to that separated set to obtain an accuracy estimate.

#### Accuracy of the Cavnar and Trenkle procedure

Based on the training set, the routine proposed by Cavnar and Trenkle was used to create a profile for each one of the nationalities, based on the 1,250 most frequent n-grams (n = 3). Table 3 displays the confusion matrix resulting from the application of the algorithm to the test data set.

**Table 3:** Confusion matrix—observed and predicted values obtained using the Cavnar and Trenkle procedure for the classification of surnames in the test set.

	Predicted					
Observed		EAS	GER	IBR	ITA	JPN
	EAS	0.67	0.17	0.10	0.03	0.03
	GER	0.07	0.86	0.04	0.03	0.01
	IBR	0.03	0.04	0.74	0.16	0.04
	ITA	0.02	0.02	0.17	0.77	0.02
	JPN	0.01	0.00	0.02	0.02	0.96

Note: share of correct guesses are located in the diagonal of the table.

The table shows that, in spite of its simplicity, the Cavnar and Trenkle algorithm proved to be reasonably accurate. In the case of the Japanese (JPN), 96% of the test data were classified correctly. The worst results were obtained for the Eastern European (EAS) surnames, where only 67% of the surnames in that category were effectively classified as such. Overall accuracy is the total number of correct predictions divided by the total number of observations. The estimated accuracy was 80.1%. The Kappa value, that is, the percentage of correct classifications, taking into account the possibility of correct classifications’ occurring at random, was equal to 0.73.

#### Accuracy of the Naïve Bayes procedure

Unexpectedly, the results obtained by applying the Naïve Bayes algorithm were worse than those obtained with the Cavnar and Trenkle algorithm (see Table 4). Although the former was more accurate in the case of German nationality, for most of the other nationalities the Bayes procedure proved to be fairly inaccurate. The worst errors were associated to the classification of the East European surnames; almost half of them were classified as if they were German. Similarly, the Naïve Bayes algorithm mistakenly classified 45% of the Iberian surnames as Italian.

**Table 4:** Confusion matrix—observed and predicted values obtained using the Naïve Bayes procedure for the classification of surnames in the test set.

	Predicted					
Observed		EAS	GER	IBR	ITA	JPN
	EAS	0.31	0.52	0.05	0.11	0.00
	GER	0.01	0.87	0.02	0.11	0.00
	IBR	0.01	0.23	0.33	0.43	0.00
	ITA	0.00	0.18	0.09	0.73	0.00
	JPN	0.00	0.15	0.04	0.08	0.72

Note: share of correct guesses are located in the diagonal of the table.

Accuracy was 68% and the Kappa value was 54%, both much poorer than the values obtained with the Cavnar and Trenkle method. Other articles describing applications of the Naïve Bayes to classify surnames in other countries have reported a much higher degree of accuracy than that obtained in the present research [5]. One possible explanation for the lower accuracy of the present algorithm is that the literature measures the accuracy considering repeated surnames, while this paper takes into account unique surnames. Furthermore, other papers that achieve greater accuracy classify surnames into fewer categories. [5] It should be borne in mind that, in spite of the classificatory algorithm’s unusually poor performance, its classification errors only affected 3.3% of the individuals in the RAIS base.

### Classification results

Given the superiority of the Cavnar and Trenkle algorithm compared to the Naïve Bayes, only the former are presented. In terms of classifying unique surnames present in the RAIS, the results are set out in column 1 of the Table 5. Those percentages do not reflect the ancestry of the population because the concentration of surnames varies from one ancestry to another. It is more interesting to examine columns 2 and 3, which present the distributions of the population by ancestries. Around 16.3% have at least one Germanic, East European, Italian or Japanese surname and 11.9% have a non-Iberian last or unique surname.

**Table 5:** Surname ancestry estimated according to the last or unique surname.

Ancestry	Acronym	(1) % of unique surnames	(2) # of individuals	(3) % of individuals
Iberian	IBR	27.4%	41,215,227	88.1%
Italian	ITA	32.4%	3,377,695	7.2%
German	GER	20.5%	1,504,894	3.2%
Eastern Europe	EAS	13.1%	392,708	0.8%
Japanese	JPN	6.6%	311,488	0.7%
Total		100%	46,802,012	100%

Note: column 1 shows the shares of each ancestry in the **unique surname** database; column 2 and 3 present number and share, in this order, of each ancestry in the **individuals** database.

#### Analysis of the geographic distribution of the surnames

An immigrant ancestry index was created for synthesis purposes. If an individual had only Iberian surnames the index was set to zero. If he had exclusively non-Iberian names the attributed index value was 1. If one of the surnames was Iberian and the other was not (irrespective of their order) the index value was set to 0.5.

**Fig 2:**
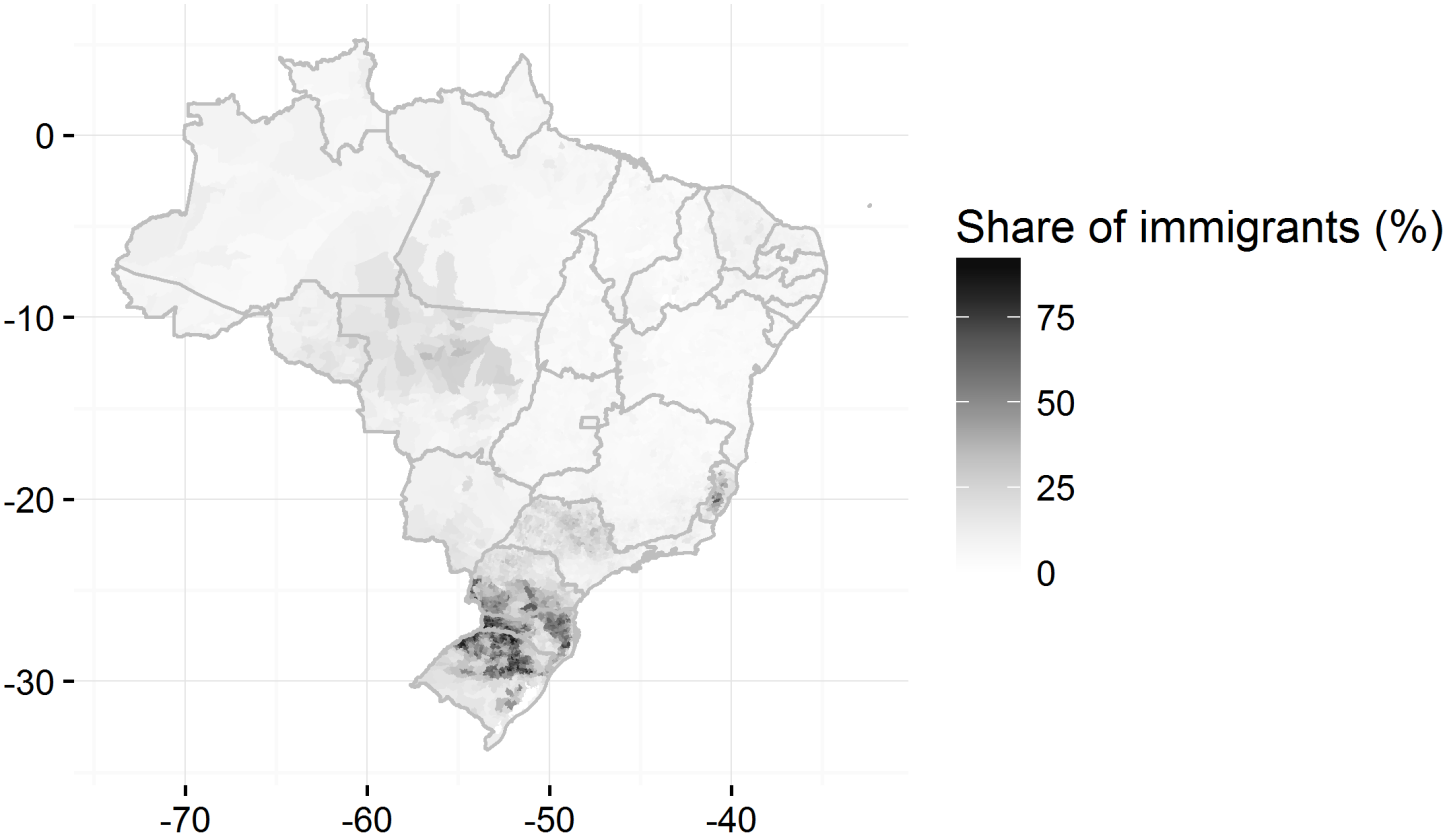
Index of non-Iberian surnames by municipality. Note: index = 1 for individuals with exclusively non-Iberian surnames; index = 0.5 for one Iberian and one non-Iberian surnames; index = 0 for exclusively Iberian surnames. Map shows the rate of the sum of the index by the number of individuals in each municipality.

Fig 2 illustrates the distribution of that index in Brazilian territory and section S1 Maps contains maps for the distribution of each non-Iberian ancestry. The maps are consistent with the literature on immigration and migration [19]. The historical record sustains that municipalities with the highest concentrations of non-Iberians are located in places that received settlements of those groups from the XIX century until the 1920s. German immigrants were the first non-Iberians to have their immigration subsidized by the Brazilian government and they founded the colony of São Leopoldo in Rio Grande do Sul in 1824 [36]. That process continued in the three southernmost states of Brazil and progressively Italians and eastern Europeans were included in both private and official immigration initiatives in those areas [37][38].

The mass immigration of Italians and Japanese to São Paulo began in the last decades of the 19th century as a bid to replace slave labor in São Paulo’s coffee-growing areas [39][40]. That is not clearly visible in the maps because in the 1930s that state became the manufacturing powerhouse of Brazil and received vast contingents of poor migrants from the Brazilian Northeast in the decades that followed.

So how can the existence of high percentages of immigrants in certain areas of the Brazil’s central-western and northern regions be explained? The states of those regions are the area into which Brazil’s agricultural frontier has been expanding and, accordingly, in the course of the last three decades, they have received thousands of farmers from the south of Brazil [41]. Japanese settlements were created in Pará and in other states of the Amazon region in the nineteen-twenties. This explains the high concentration of japanese surnames in some municipalities [42]. As a result non-Iberian surnames have become quite frequent in areas that were previously only sparsely populated.

These results suggest that the process of classification based on fuzzy matching complemented by the Cavnar and Trenkle algorithm correctly identified the ancestry of the workers registered in the RAIS.

**Fig 3:**
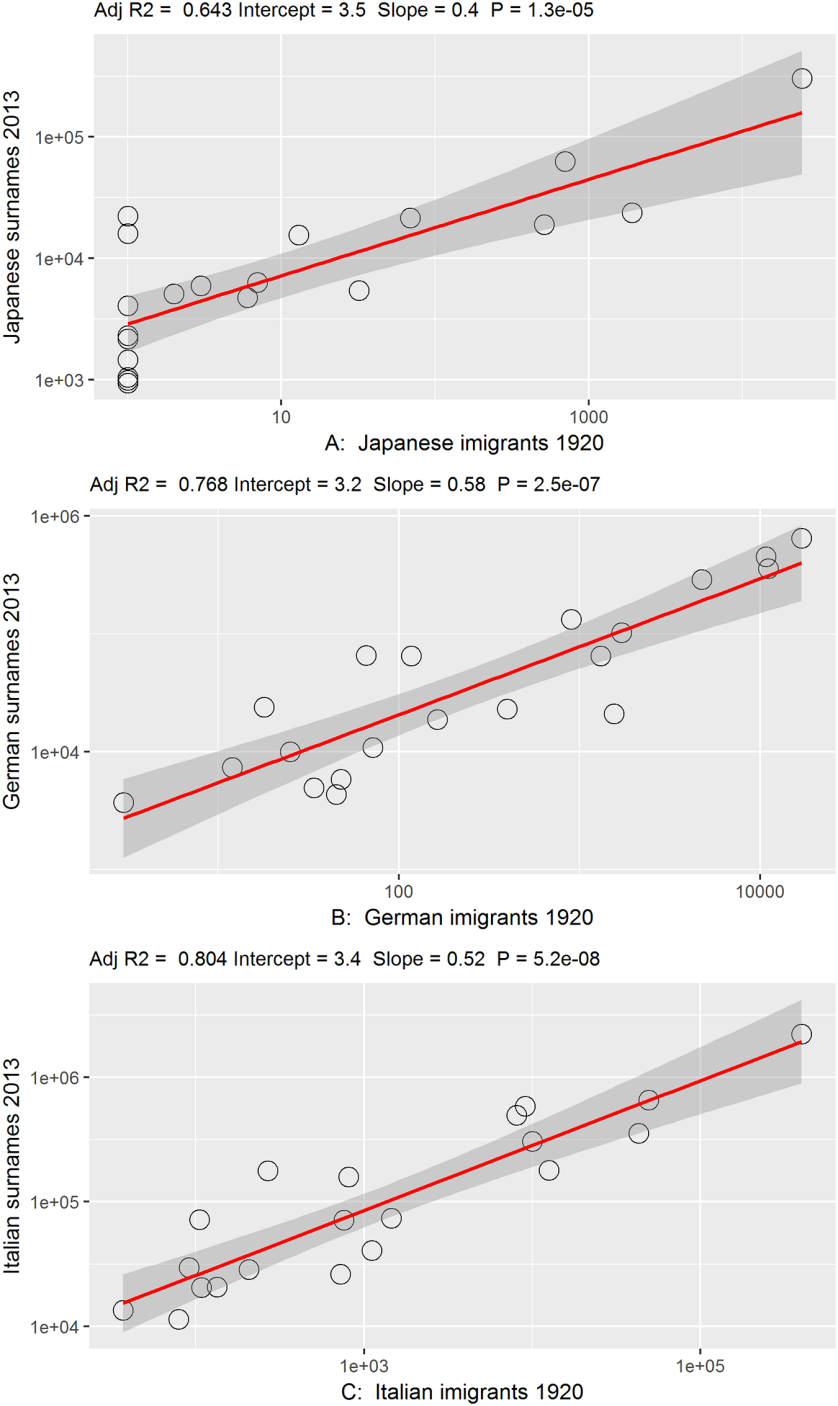
Number of foreigners in 1920 Census and Brazilians with non-Iberian surnames in 2013 by state. A: Japanese. B: German. C: Italian. Note: graphs in bi-logarithmic scale. Eastern Europeans were omitted because in the 1920 Census they were classified in the category “Others”.

Another way to assess the external validity of the algorithm is to compare it to the data on foreigners in the 1920 census, made at a time when the last large wave of immigration to Brazil had just ended. Fig 3 shows the correlation between the number of foreigners in Brazil at that time and the totals of those that were identified as descendants of Japanese, Italians, and Germans, distributed by states. Contemporary data was aggregated in order to follow 1920 state limits and the scatter plot of Iberians were not been presented because, as they are such a large contingent, the illustration would merely reflect changes in the state population from 1920 to 2013. The correlations show that in spite of the recent internal migrations, including those associated to the expansion of the agricultural frontier, the descendants tend to reside in the same state as their forebears. That result also inspires greater confidence in the surname classification procedure, especially at the level of aggregated data.

#### Wage and schooling by ancestries

In order to obtain a broad vision of Brazilian workers, the following reclassification was made by combining the official data on color/race with the estimated values. *The class obtained using the surname classification algorithms was used, except when the individuals in question were registered in the RAIS database as being “black”, “mixed” or “native”. In such cases the original classification was maintained*. In that way 8 categories of ancestry/skin color were obtained, namely, Iberian (IBR), Japanese (JPN), Italian (ITA), German (GER), Eastern European (EAS), black (BLK), mixed (MXD) and native (NAT).

**Fig 4:**
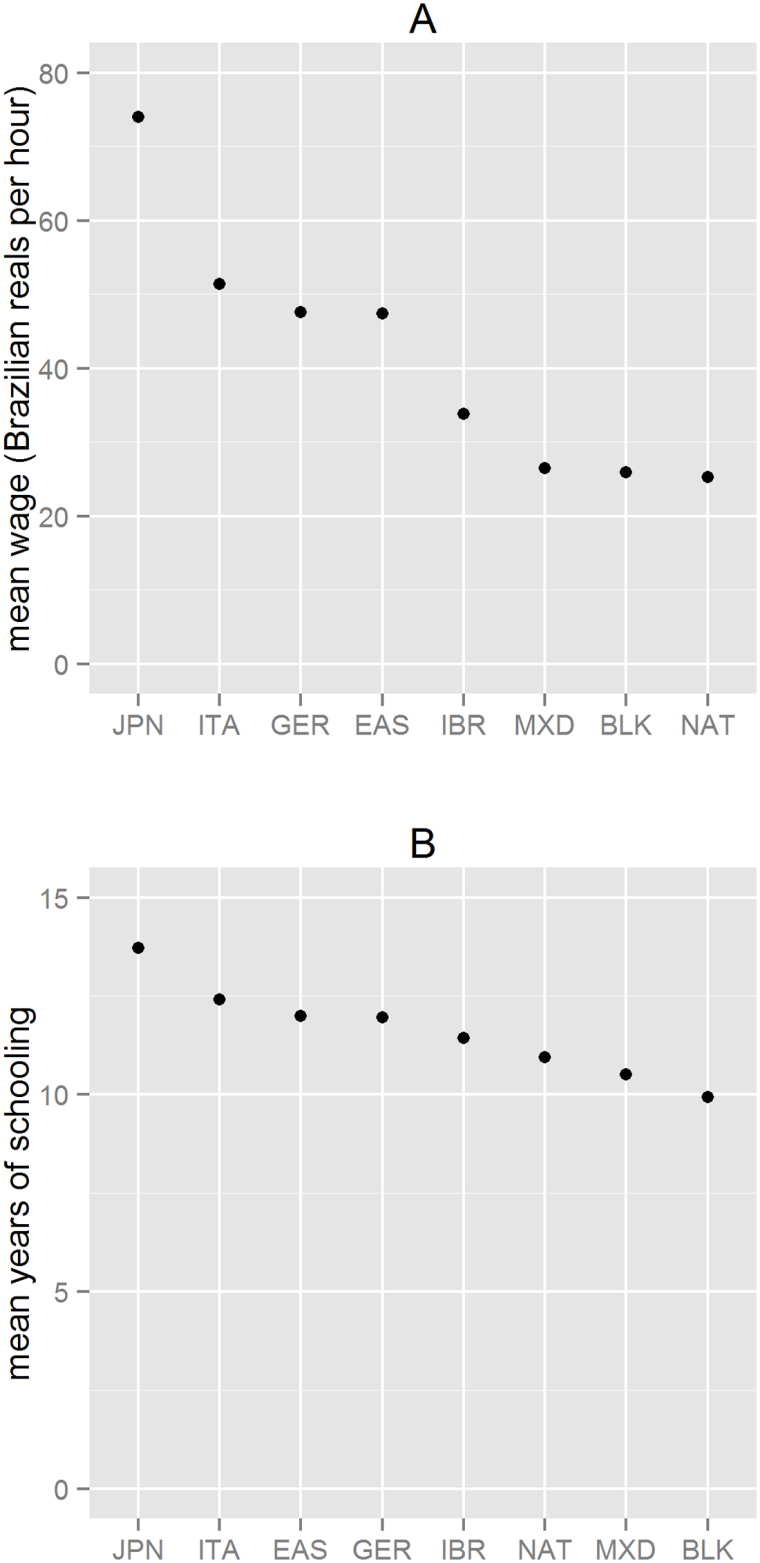
Wage and schooling by surname ancestry/color in Brazil (2013). A: Average hourly wage (in Brazilians Reals). B: Average schooling (in years). Note: data on levels of schooling was converted to years of schooling.

Fig 4 is descriptive and not causal. Only Brazilians in the age group 23 to 60, working in the private sector at least 40 hours a week were selected. It is well known that the lowest wages and schooling are found among Brazilian workers whose color/race is registered as black, mixed, or native. However the figures also reveal that individuals with non-Iberian surnames earn salaries substantially higher than those registered as white, with Iberian surnames. Those with Japanese and Eastern European ancestry earn, on average, R$73 and R$52 an hour, whereas for Iberians the amount is less than R$34 an hour. The differences of schooling levels are substantial but not so great. Japanese and Italian ancestries have an average of 13.6 and 12.4 years of schooling respectively, compared to 11.4 years for Iberians. All differences in schooling and wages by ancestry are statistically significant at 99%.

Table 6 presents the results of the Two- Way ANOVA of wages by surname ancestry-color with years of schooling as covariate. In Brazil, education discrepancies are huge and returns for schooling are high [43], so it is not surprising that the analysis yielded a main effect for levels of schooling. Nevertheless, ancestry-color categories were statistically significant at very strict levels significance levels. Interaction effects between schooling and ancestry-color are statistically significant as well.

**Table 6:** Hourly wages in Brazil (2013)- Analysis of variances.

Variable	Sum of Squares	Df	F-value	Pr(>F)
Levels of schooling	160649726	10	8013.606	<2.2e-16
Ancestry-color	6035069	7	430.063	<2.2e-16
Interaction schooling and ancestry	7122734	69	51.493	<2.2e-16
Residuals	876087185	437014		

Note: type II tests.

Various economic, sociological and even geographic factors can explain salary and schooling differences, but that is a discussion outside the scope of the present paper. In any event, ancestry classification of surnames revealed differences that would have remained hidden if the usual color/race classifications had been the only ones considered.

## Conclusion

This paper has shown the potential of using historical sources and contemporary fuzzy matching and machine learning techniques to classify the Brazilians by their surname ancestry.

The classification process was fairly accurate. Even thought the accuracy of the Cavnar and Trenkle algorithms and, even more so, the Naïve Bayes algorithm were poorer than had been expected, that does not appear to have jeopardized the overall results. After all, the great majority of individuals was classified by fuzzy matching and only 3.6% by the Caviar and Trenkle procedure. Furthermore, the geographic distribution of workers with non-Iberian surnames at the municipal level and at state level as well reflect both historical and contemporary knowledge about the initial location of immigrants and internal migrations.

In Brazil, 16.3% of the workers have at least one Germanic, East European, Italian or Japanese surname. Spatially there are states with very high concentrations of non Iberian names and they generally coincide with the 1920 Census data on foreigners in Brazil. It also became very clear that the ancestry of the surname is associated to the substantial differences in salary and schooling levels.

Obviously, it is not possible to guarantee that the classification reflects their cultural or genomic ancestry. Even when both of the individual’s surnames are used (when that is the case), there is an inevitable loss of the matrilineal lineage and, furthermore, adoptions, name changes when marrying and other events can contribute to reducing the accuracy of that particular indicator. In the case of more aggregated data, however, those idiosyncrasies tend to cancel one another and the overall trend is towards enhanced precision.

There are studies that analyze the question of self identification in regard to color/race and genetic ancestry in Brazil [44][45][46][47]. In the future, combining that literature with the analyses of the ancestry of Brazilians’ surnames could eventually shed light on a series of social, economic and public health issues.

## Supporting information

S1 Appendix Reference table data sources. (PDF)

S2 Appendix First names and surnames. (PDF)

S1 Maps Share of non-Iberian surnames. Note: index = 1 for individuals with exclusively non-Iberian surnames; index = 0.5 for one Iberian and one non-Iberian surnames; index = 0 for exclusively Iberian surnames. Map shows the rate of the sum of the index for each ancestry by the number of individuals.
(TIFF)

S1 Data Reference table (training set). (CSV)
