# Supplementary material for: Surnames and ancestry in Brazil
Source: PLoS One. 2017 May 8;12(5):e0176890. doi: 10.1371/journal.pone.0176890 (PMC5421764; doi:10.1371/journal.pone.0176890)
Supplement: S1 Appendix — (PDF) [file pone.0176890.s001.pdf]

Certain precautions were necessary in the process of constructing the reference file based on historical and contemporary sources:

- The 1880 and 1910 US Censuses [18]: People born in Germany, Poland, Italy, Russia, Hungary, the Czech Kingdom, Spain, Portugal and Japan were selected. The number of registrations is so great that it was possible to adopt more rigorous criteria for surname selection. Only surnames registered for more than eight individuals were considered;

- Fragoso and Ferreira [23]: This is a database containing the names of persons responsible for transporting slaves in the southeast of Brazil in the first half of the 19th century. Historical evidence suggests that they were Portuguese or descendants of Portuguese;

- Museu da Imigração [22]: Registers of the São Paulo Immigrants Hostel (Hospedaria dos Imigrantes) for the years 1882 to 1930. The Hostel was where newly arrived immigrants were accommodated until they moved on to their final destinations. The data was obtained by web scraping;

- Common Japanese Surnames [24]: A digital file containing contemporary surnames. To avoid the inclusion of foreign surnames, only the most frequent 4,000 were considered. The surnames in Kanji characters were converted to Roman alphabet using a Kanji converter (http://nihongo.j-talk.com/);

- Heraldica de Apellidos Españoles [25]: Webpage with traditional Spanish names ;

- Frecuencias de apellidos [26] Digital file containing the frequencies of contemporary Spanish surnames. As in the case of the Japanese names, to avoid including foreign names in the list only the most frequent 500 were considered. The names MOHAMED, BOSCH, SINGH, COLL, CHEN and WANG were manually excluded as they were obviously not typically Spanish.

- Banco de sobrenomes – cidadania italiana [27] and Emigrazione Veneta [28]: Both are databases with the names of Italian immigrants who arrived in Brazil. They were elaborated by companies that assist Brazilians who wish to acquire Italian nationality. The second database specializes in immigration from the Italian region of Veneto.

As explained in the text, for each database only those surnames for which 90% of those who bore them had the same nationality were included. Furthermore, in cases of any conflict between name and nationality, the information obtained from the Museu de Imigração records was given priority.
