# Supplementary material for: Surnames and ancestry in Brazil
Source: PLoS One. 2017 May 8;12(5):e0176890. doi: 10.1371/journal.pone.0176890 (PMC5421764; doi:10.1371/journal.pone.0176890)
Supplement: S2 Appendix — (PDF) [file pone.0176890.s002.pdf]

The frequency of first names consisting of 2 or even 3 main words required a procedure to distinguish them from the surnames. That procedure was as follows:

1. Based on the RAIS data, the 500 most frequent first names in Brazil were calculated;

2. The first word of all the first names were excluded;

3. Given that, generally, the compound first names are formed using the most popular single first names ("Maria Fernanda", "João Paulo", among so many others), the names obtained in step 1 were excluded when they were the first word of those strings obtained in step 2.

4. The surname of the individual was considered to be the last and last but one (when it existed) of the words of the string obtained in step 3. The name "José Eduardo Silva", for example, loses the first word in step 2 and the second word in step 3 so that the surname would be considered to be "Silva". The same procedure applied to "José Santos Silva" generates two surnames because"Santos" is not on the list of the 500 most popular first names.

In addition to those steps the words "Socorro", "Auxiliadora", "Dores", "Rosário", "Ribamar" were excluded because those are common second names but are not among the 500 most common first names.
