# Supplementary figures and images for: Surnames and ancestry in Brazil
Source: PLoS One. 2017 May 8;12(5):e0176890. doi: 10.1371/journal.pone.0176890 (PMC5421764; doi:10.1371/journal.pone.0176890)

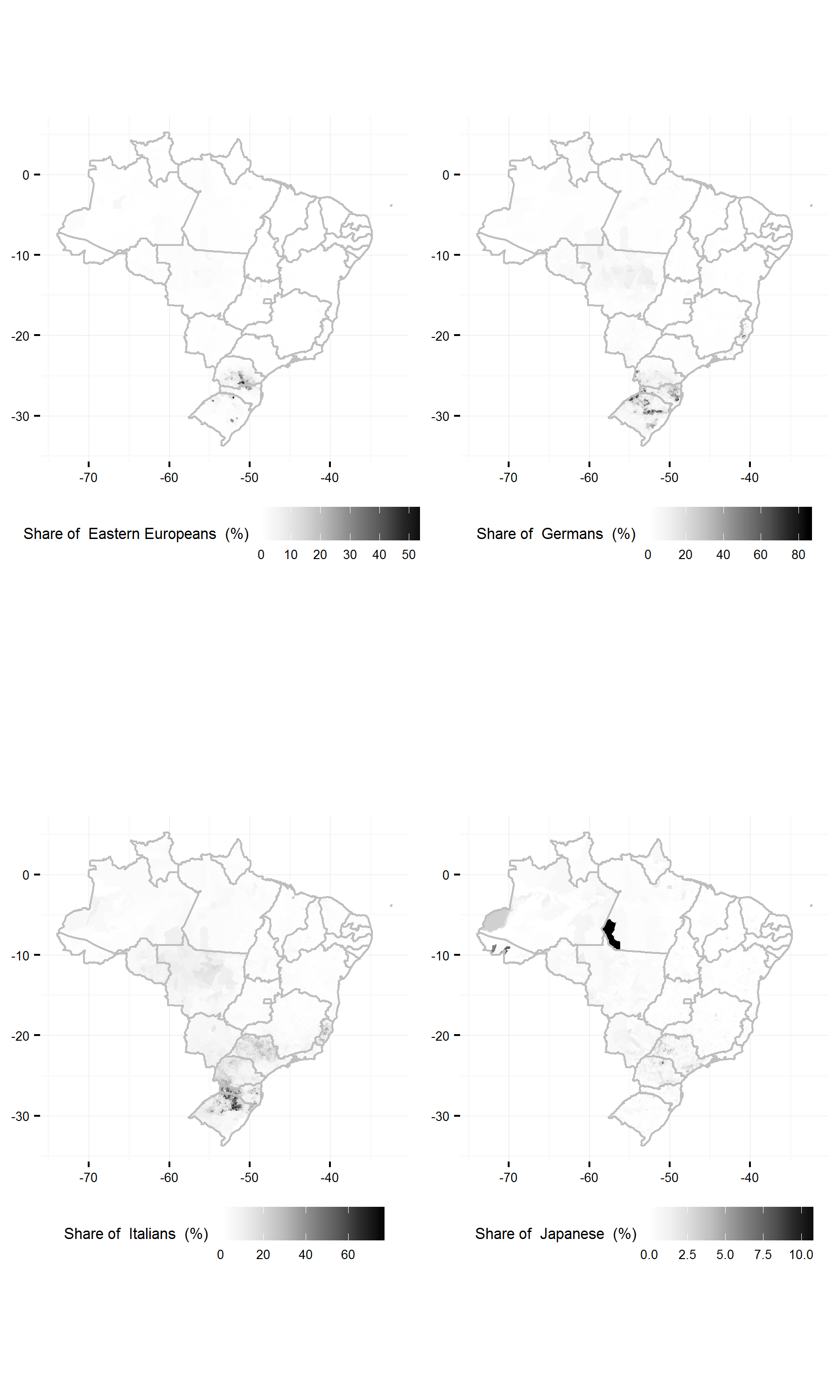

Supplement: S1 Maps — Note: index = 1 for individuals with exclusively non-Iberian surnames; index = 0.5 for one Iberian and one non-Iberian surnames; index = 0 for exclusively Iberian surnames. Map shows the rate of the sum of the index for each ancestry by the number of individuals. (TIFF) [file pone.0176890.s003.tiff]
